# Global Population Structure of a Worldwide Pest and Virus Vector: Genetic Diversity and Population History of the *Bemisia tabaci* Sibling Species Group

**DOI:** 10.1371/journal.pone.0165105

**Published:** 2016-11-17

**Authors:** Margarita Hadjistylli, George K. Roderick, Judith K. Brown

**Affiliations:** 1 Department of Environmental Science, Policy, and Management, University of California, Berkeley, California, United States of America; 2 School of Plant Sciences, The University of Arizona, Tucson, Arizona, United States of America; Chinese Academy of Agricultural Sciences, CHINA

## Abstract

The whitefly *Bemisia tabaci* sibling species (sibsp.) group comprises morphologically indiscernible lineages of well-known exemplars referred to as biotypes. It is distributed throughout tropical and subtropical latitudes and includes the contemporary invasive haplotypes, termed B and Q. Several well-studied *B*. *tabaci* biotypes exhibit ecological and biological diversity, however, most members are poorly studied or completely uncharacterized. Genetic studies have revealed substantial diversity within the group based on a fragment of the mitochondrial cytochrome oxidase I (mtCOI) sequence (haplotypes), with other tested markers being less useful for deep phylogenetic comparisons. The view of global relationships within the *B*. *tabaci* sibsp. group is largely derived from this single marker, making assessment of gene flow and genetic structure difficult at the population level. Here, the population structure was explored for *B*. *tabaci* in a global context using nuclear data from variable microsatellite markers. Worldwide collections were examined representing most of the available diversity, including known monophagous, polyphagous, invasive, and indigenous haplotypes. Well-characterized biotypes and other related geographic lineages discovered represented highly differentiated genetic clusters with little or no evidence of gene flow. The invasive B and Q biotypes exhibited moderate to high levels of genetic diversity, suggesting that they stemmed from large founding populations that have maintained ancestral variation, despite homogenizing effects, possibly due to human-mediated among-population gene flow. Results of the microsatellite analyses are in general agreement with published mtCOI phylogenies; however, notable conflicts exist between the nuclear and mitochondrial relationships, highlighting the need for a multifaceted approach to delineate the evolutionary history of the group. This study supports the hypothesis that the extant *B*. *tabaci* sibsp. group contains ancient genetic entities and highlights the vast cryptic diversity throughout the genome in the group.

## Introduction

The evolutionary processes leading to speciation in ecologically and genetically divergent populations have attracted the interest of many [1–3], leading to a focus on the study of speciation in phytophagous insects [4–7]. One view of speciation is that of a continuous process involving polymorphic populations as they evolve to become distinct species, with “biotypes” or “ecological races” acting as intermediate stages [5, 8]. At the other extreme, there are several empirical examples of morphologically conserved lineages, apparently reproductively isolated for millions of years that are either allopatric (e.g. [9, 10]) or have shifted into sympatric (e.g. [11]) or parapatric ranges (e.g. [12]). Such populations of morphologically conserved lineages that are genetically divergent and often also reproductively isolated, are referred to as cryptic or sibsp. because of their previous classification into a single taxon, based on identical morphologies. Cryptic species are more common than previously expected, and are now known to occur across major metazoan taxa and biogeographical regions [13]. With advances in molecular and genetics approaches the discovery and description of cryptic species have increased exponentially in the past two decades [14].

The view that cryptic lineages are the outcome of recent speciation events has been contested in light of studies suggesting ancestral divergence of morphologically cryptic lineages, in some cases dating to the Oligocene i.e. 24 million years ago ([10, 14, 15] and references therein). It has been suggested that behavioral, physiological, and developmental plasticity may allow organisms to compensate for environmental perturbations without requiring morphological change [16], a collection of mechanisms that when invoked lead to “morphological stasis”. Therefore, persistent morphologies can be maintained by stabilizing selection [17], while divergence at other traits (behavioral, ecological, genetic) and ultimately speciation can proceed at a “normal” pace. Such selection pressures may be imposed especially by conditions experienced at extreme or ‘marginal’ environments [14].

Studies of multiple co-distributed cryptic lineages, combining phylogeographic and population genetics approaches provide an excellent framework to appreciate cryptic biodiversity [15]. One such system is the whitefly sibling species (sibsp.) group *Bemisia tabaci* Grennadius (Hemiptera: Aleyrodidae) for which the taxon was first described as *Aleyrodes tabaci* in 1889 (see references in [18]). This group comprises an untold number of cryptic lineages worldwide [18–23], some of which currently overlap in geographic range. The morphologically indistinguishable lineages, traditionally characterized as *B*. *tabaci* biotypes [18, 22, 24] with many distinguished more recently as mitochondrial (COI) haplotypes show variability in certain biological and ecological traits, among which are plant virus transmission efficiency (competency), insecticide resistance, fecundity, dispersal, and mating behavior [24]. In addition a few are invasive pests while all are vectors of the genus, *Begomovirus* (*Geminiviridae*), and most typically are thought to be polyphagous, with a small number of monophagous types, as well as types that may be restricted by host and geographic ranges [18, 22, 24]. Ever since molecular techniques were used initially to study this system in the late 1980’s, it became evident that this complex also exhibits extreme variation at the genetic level (for reviews see [23, 24, 25]). Recent analyses of a comprehensive mtCOI dataset, some of which are available in the GenBank database, recognized a large number of cryptic variant groups, or haplotype clades [19, 24]. Using similar datasets Dinsdale et al. [20] and Lee et al. [26] proposed the treatment of entities within the dataset as operational taxonomic unites (OTUs), and a hypothetical ‘species’ cutoff at 3.5% or 4.0%, respectively, based on mtDNA divergence only (see designations in S1 Table), which excludes consideration of phylogenetic relationships or biological characteristics. The particular ecological factors that have contributed to the apparently extreme diversification in *B*. *tabaci* are not yet understood, but it has been suggested that lineages of *B*. *tabaci* diverged millions of years ago following separation of continental landmasses [22], coinciding with a period of global diversification across the plant and animal kingdoms that were associated with major climatic and tectonic events [27]. Evidence supporting this hypothesis stems from the unexpectedly high divergence between mtCOI sequences among clades-up to ~26% [24], and phylogeographic separation among many mtCOI gene clades [20].

Understanding the boundaries within this species group ideally requires a thorough assessment of different biological and ecological characters, together with mating experiments, to determine which lineages are reproductively isolated and/or to what extent. However, establishing which lineages have developed reproductive barriers, thus constituting “distinct gene pools”, can be assessed through indirect estimates of gene flow by studying differentiation in multiple nuclear markers [28]. Genetic analyses not only provide information about contemporary gene flow among lineages, but also shed light on historical demographic processes such as ancestral population expansions and time of divergence from a common ancestor.

Analyses with several markers and DNA sequences of a number of gene regions in *B*. *tabaci* have provided us with a general picture of the biogeographic distribution of lineages, with the mtCOI gene being the most diverse and other nuclear loci showing much less divergence [29, 30]. Thus, our current view to date of the global phylogenetic and phylogeographic relationships in *B*. *tabaci* relies solely on the mtCOI [19, 20, 22, 24, 30]. Although this marker has been widely used to assess the divergent and cryptic nature of the *B*. *tabaci* sibsp. group its overall value for informative phylogeographic reconstruction, species boundaries delineation, and understanding population structure has been strongly criticized [31–35]. Issues include, (1) that inheritance of mtDNA is matrilineal and thus not representative of processes involving males, (2) the mitochondrial genome is small but also contains a large proportion of functional genes and thus likely to be impacted by selective sweeps [34, 36], (3) mtDNA is known to move between species in many organisms [37], and (4) for stochastic reasons, as one locus it may not be representative of the actual demographic history of the organism. Indeed, in *B*. *tabaci*, this marker exhibits 0–26% divergence [24], which exceeds the variation typical of closely related species that may be attributable to effects of haploid males and/or bacterial symbionts [38]. For these reasons divergence of mtDNA itself is not sufficient for describing species or for understanding the history and structure of biological diversity.

Microsatellite markers are used for the inference of genetic relationships between populations of the same species or closely related species because of their high polymorphism owing to high mutation rates and representation across the nuclear genome [39]. Multiple microsatellite loci have been isolated from *B*. *tabaci* [40–46] and have been used to examine local population structure and differentiation in biotypes (for a review see [25]), providing insights into the roles of host and geography in structuring of populations [47–49], in detecting hybridization among sympatric invasive and indigenous biotypes in a region [42], in detecting associations of biotypes with differential insecticide resistance levels [50] or endosymbiont composition [50–52] and in identifying the sources and routes of dispersal of invasive biotypes in new areas [25]. However, multiple nuclear loci have not yet been employed to determine the genetic structure of worldwide lineages and biotypes.

In this study, we used 13 microsatellite loci to resolve the global population structure of the *B*. *tabaci* sibsp. group and add further insight into the current relationships within and between *B*. *tabaci* lineages. The goal was to determine whether “biotypes” that were initially described on the basis of esterase markers and then based on mtCOI analysis, together with biological and ecological data, are also phylogenetic entities, that is, whether they form monophyletic groups with restricted, or perhaps, no gene flow among them. By analyzing the worldwide distribution of populations representing monophagous, polyphagous, widespread invasive, and indigenous biotypes, we aimed to examine not only the global evolutionary history, but also differential patterns of genetic diversity across this sibsp. group. Finally, we compared our results to published mtCOI phylogenetic conclusions to determine what additional insights these nuclear genome markers might provide to illuminate the diversity, population structure, and history of *B*. *tabaci*.

## Materials and Methods

### *Bemisia tabaci* samples and populations

A total of 839 female whiteflies from 50 collections, sampled worldwide were genotyped in this study (S1 Table). A permit was not required from the country of origin for whitefly collections because *B*. *tabaci* is an agricultural pest widely distributed in the field and plantations/greenhouses, not a species of any conservation status. Whiteflies were collected from both public and private land. In cases where collection was done from private land, permission to collect was granted by landowners. Adult females were used for genetic analysis because whiteflies are haplodiploid with diploid females and haploid males [53]. The samples span distinct geographic sampling locations representative of the global distribution of *B*. *tabaci* and were obtained through direct field sampling and laboratory collections (J.K. Brown and many collaborators, worldwide) (S1 Table). The field samples encompassed partially characterized or uncharacterized haplotypes, and well-studied biotypes, collectively, representing haplotypes that cluster in six (or seven) major clades inferred by the mtCOI phylogeny of the sibsp. group [24], and sequences considered to be separate species by Dinsdale et al [20] and Lee et al [26].

### DNA extraction

Whitefly adults were collected live and preserved in 95% ethanol. Whiteflies from most collections (adults, with nymphs when available) were identified to species by R. Gill (CDFA, Sacramento, CA). Samples that originated from previously studied or biotype reference collections have been assigned to mtCOI clade (S1 Table), based on previously sequenced data at the mtCOI gene. For newly studied collections a region of approximately 800 bp of the mtCOI gene was amplified and sequenced in both directions for at least one specimen per population using primers and protocols described in Hadjistylli [54] to confirm that these belong to the *B*. *tabaci* sibsp. group and to identify the mtCOI clade they belong to. GenBank accession numbers for all available sequences are provided in S1 Table. For DNA extraction, whole adult whiteflies were homogenized in individual 1.5 ml microcentrifuge tubes and genomic DNA was extracted using the Qiagen DNeasy DNA Blood and Tissue kit following the manufacturer’s protocol. A modification to this protocol was implemented to perform a final elution of the DNA from the column in 80μl buffer AE, followed by a second elution in 20μl AE, with the two eluates combined in a single microcentrifuge tube and stored at -20°C.

### Microsatellite loci

A total of 13 microsatellite loci (Table 1) were amplified using polymerase chain reaction (PCR) (see protocols below). We used loci developed in our laboratory [44] and from several literature sources [40, 41, 43, 45, 46]. The loci we used were isolated from different *B*. *tabaci* biotypes and had different repeat motifs, likely evolving at different rates (Table 1). Out of a total of 65 microsatellite primer pairs we tested and screened, we selected 13 loci that cross-amplified in most of our populations. Samples that failed to amplify at any given locus were genotyped at least 3 times before scoring those genotypes as missing data.

**Table 1:** Characteristics and sources of microsatellite loci used in this study.

Microsatellite locus	Allele size range (bp)	Fluorescent dye color^c^	Repeat motif	Isolation source	Reference
^a^**WF1B11**	101–178	PET	(CCTGA)_12_imp^d^	Biotype B	[44]
**WF2C01**	110–225	PET	(GTTT)_11_imp	Biotype B	[44]
**WF2H06**	141–214	NED	(TTTG)_11_	Biotype B	[44]
**WF1B06**	128–170	PET	(ACTC)_8_	Biotype B	[44]
**WF2E11**	159–264	PET	(GATT)_27_imp	Biotype B	[44]
^a^**BEM6**	161–236	6-FAM	(CA)_8_imp	Australia	[41]
^a^**BEM15**	166–238	6-FAM	(CAA)_6_(CAG)_4_(CAA)_4_	Asia (Indonesia)	[41]
^a^**BEM31**	105–142	HEX	(GCT)_4_(GTT)_2_	Asia (Vietnam)	[41]
^a^**BT-b103**	118–151	HEX	(AC)_8_(TC)_3_	Biotype Q	[45]
^a^**BT-e49**	266–390	6-FAM	(TTG)_12_(TTC)_11_	Biotype Q	[46]
**BtIs1.2**	256–368	6-FAM	(CA)_13_N_14_(CA)_8_	B/Q	[43]
^b^**MS145**	171–225	PET	(AC)_9_	Biotype B	[40]
^a^**MS177**	233–278	6-FAM	(CA)_7_	Biotype B	[40]

^a^Denotes loci used in the 7-locus analysis

^b^Denotes additional locus used in the PCA

^c^6-FAM, PET, NED (Applied Biosystems), 6-FAM, HEX (SIGMA-ALDRICH)

^d^Imperfect

### PCR protocols and microsatellite genotyping

Microsatellite forward primers were labeled with a fluorescent dye with an unlabeled reverse primer (Table 1). PCR was carried out as described in Hadjistylli et al. [44]. When it was possible to assess the allelic range for each population, subsequent amplifications of loci across all populations were carried out using multiplexing multiple primer pairs in a single reaction (3–5 pairs per reaction, labeled with different fluorescent dyes, or having discrete allele size ranges). Multiplex PCR was done in 96-well plates using the Multiplex PCR Kit (Qiagen) in 15 μl reaction volumes containing 6 μl of the Qiagen PCR Master Mix (1X), 2 μM of each primer, 3 μl RNase-free water and 1 μl of genomic DNA. Thermocycling conditions were as follows: 15 min at 95°C followed by 30 cycles of 30 s at 94°C, 90 s at 60°C, 60 s at 72°C, with a final extension step of 60°C for 30 min. Final PCR products were mixed with a cocktail of 48:1 Hi-Di formamide (ABI): LIZ500 size standard (ABI) (0.5 μl PCR product, 0.2 μl LIZ, 9.3 μl formamide) and were denatured at 95°C for 5 min. Fragments were ran on an ABI 3730 DNA sequencer and genotypic data were visualized and scored manually using the software GeneMapper version 4.0 (ABI). All runs included negative and multiple positive experimental controls to ensure consistency in allele scoring. The program Flexibin [55] was used to facilitate binning and help detect miscalled microsatellite alleles.

### Basic population genetics statistics

Prior to any analyses we tested for significant differentiation among populations sampled from neighboring locations using the exact test as implemented in Genepop (version 4.0.10) [56, 57]. Significance levels were corrected for multiple comparisons using the standard Bonferroni correction at the 0.05 levels. Populations that were not significantly differentiated were pooled into a single sample for further analyses (see S1 Table). We used the program GenAlEx 6.1 [58] to calculate locus-specific statistics (number of alleles, total expected heterozygosity, mean expected and observed heterozygosities and *F* statistics) across all populations as well as population specific statistics across all loci and per locus (Na, *H*_O_, *H*_E_, and *F* statistics).

Because not all of our samples amplified at all loci, to obtain unbiased estimates of genetic diversity we used a reduced dataset based on 7 loci (see Table 1), excluding any individuals or whole populations that had any missing data (e.g. Uganda-sweetpotato, China-Hainan, Jatropha-Puerto Rico (PR), Guatemala, Mozambique), resulting in a reduced dataset with 690 individuals. Loci isolated from different biotype sources were selected for these calculations e.g. two loci from biotype B, two from biotype Q, two from Asian populations, one from an Australian population, to avoid bias associated with primer development (e.g., microsatellites were more variable in the biotype from which they were isolated). Observed (*H*o) and expected (*H*_E_) heterozygosities per population were calculated in GenAlEx 6.1. For presentation purposes *H*o and *H*_E_ were averaged across populations for each biotype, haplotype, or geographic group based on the results of the neighbor joining tree (NJ) and clustering analyses (explained below). Allelic richness (number of alleles) was calculated using the rarefaction method implemented in the program HPrare [59] to correct for differences in the size and number of populations per biotype / region. Rarefaction standardized samples to the minimum eight genes per population and one population per region. We used the hierarchical sampling scheme offered in HPrare to group populations into known biotypes or geographic regions as determined from the NJ tree. Allelic richness between pairs of populations was then compared using analysis of variance with loci considered as blocks using a randomized complete blocks design, followed by Tukey’s multiple comparison tests in R [60].

Tests for significant genotypic linkage disequilibrium (LD) among all pairs of loci and for significant deviations from Hardy-Weinberg equilibrium (HWE) were conducted in the program Genepop (version 4.0.10) using the Markov chain method with default settings. The significance levels were adjusted using a Bonferroni correction (0.05 level).

### Null alleles

Null alleles can potentially bias estimates of genetic differentiation by reducing the genetic diversity within populations thereby increasing and overestimating the inter-population genetic differentiation (estimates of *F*_ST_ and genetic distance) [61, 62]. In order to address this issue we used the program FreeNa [61] in our full dataset to calculate unbiased global and pairwise *F*_ST_ of Weir [63] and Cavalli-Sforza and Edwards chord distance (*D*_*C*_) [64] corrected for null alleles using 1000 bootstrap replications over all 13 loci. Pairwise *D*_*C*_ corrected and uncorrected for null alleles were used to construct neighbor-joining (NJ) trees using *Neighbor* available in the software package Phylip-3.69 [65]. Trees were used for comparing the extent to which null alleles could bias the other analyses reported here. The estimated frequency of null alleles per locus for each population was calculated in FreeNa using the EM algorithm [66].

### Individual-based analyses

#### Neighbor Joining tree

The program Populations version 1.2.30 [67] was used to construct an unrooted NJ tree, based on *D*_*C*_ among individuals using information from the full dataset. *D*_*C*_ is less biased by the presence of null alleles compared to Nei’s [68] standard genetic distance (*D*_*S*_) [61]. In addition, *D*_*C*_ does not make the assumption of constant population size or constant mutation rates among loci and performs better than other genetic distances in recovering correct tree topologies [69]. The NJ analysis was done at the individual rather than at the population level to allow for the detection of genetic structure in sample populations and also for the potential migration of individuals between populations. The output obtained from the program Populations was visualized using the Interactive Tree Of Life (iTOL) tool available on line at http://itol.embl.de/ [69–71].

#### Principal Coordinates Analysis (PCA)

Principal coordinates analysis (PCA) was used as an alternative analysis of the individual genotypes to compare the consistency of results between methods. It allows for detection of the major patterns in a multivariate dataset, such as a microsatellite dataset with multiple samples and loci [58] by transforming and condensing the multilocus genotype information into a smaller number of derived variables. The Excel based program GenAlEx 6.1 [58] was used to calculate a genetic distance matrix, using option codominant-genotypic, method described in Smouse and Peakall [72] and to convert this into a covariance matrix with data standardization for the PCA. The genetic distance matrix estimation is based on a pairwise individual-by-individual calculation without taking into account the population that an individual belongs to. For a simpler display of individuals from all populations into a single PCA we color-coded individuals belonging in the same biotype or geographic group (as determined from the NJ tree). For this analysis the seven loci that amplified across most populations and an additional locus (Table 1) were used, as well as individuals having few missing data that added valuable information to the PCA, resulting in a dataset with 712 individuals.

#### Bayesian clustering analysis to assess worldwide population structure

To identify major genetic clusters in the worldwide populations of *B*. *tabaci* we used the Bayesian clustering approach implemented in the program Structure 2.3.3 [72–76]. Since the model used in this method assumes Hardy-Weinberg and linkage equilibrium within populations we excluded the three loci that significantly deviated from HWE in more than one population out of 41 (S2 Table). No population was excluded from the analysis, because no consistent pattern of deviation from HWE was evident for multiple loci. We ran five replicates, each using a burn-in length of 100,000 and a run length of 1,000,000 steps, with the admixture and the correlated allele frequencies models since some of the populations are likely admixed and have shared allele frequencies, without using prior population information (geographic sampling location).

Because after multiple runs using STRUCTURE, the strongest signals of genetic partition were observed among very divergent genetic groups, while at the same time structure could not be detected at lower levels of differentiation, the data set was subdivided to examine substructure within each of the inferred clusters. For the Q biotype specifically, because the global STRUCTURE analysis indicated the presence of two geographic genetic clusters within the biotype, we ran three different analyses at the substructure level to further examine differentiation within these clusters: one run with all Q biotype populations and two separate runs, for each geographic cluster of the Q biotype.

For each of these sub-structure runs, loci that contained missing data or that deviated from Hardy-Weinberg equilibrium for those populations were excluded. Each run comprised 500,000 iterations following a 100,000 burn-in period, for 3 replicates, and with the admixture and correlated allele frequencies models. The initial number of clusters (*K*) to be tested for each run is given below each of the STRUCTURE plots. To determine the best *K* value explained by the data for all runs the posterior probabilities were examined for each *K* and the Δ*K* estimator, as described by Evanno et al. [77] using the program STRUCTURE HARVESTER [78] (S1 Fig). Results from replicates for the inferred *K* from each run were analyzed in the program Clumpp [79] to produce averaged matrices of individual and population cluster membership coefficients. Finally, the program Distruct v1.1 [80] was used to produce graphical displays of the resulting bar plots. The program Baps [81] was used as an alternative Bayesian clustering approach to compare the consistency of these results with those obtained using Structure.

Genetic differentiation within the two invasive biotypes B and Q was further examined with analysis of molecular variance (AMOVA), a statistical procedure that allows the hierarchical partitioning of genetic variation among populations and regions [82, 83], using the program GenAlEx 6.1 [58]. We used the estimate *Φ*_*PT*_, an analogue of *F*_*ST*_ that calculates population differentiation based on genotypic variance by suppressing intra-individual variation, which is a more suitable estimate for comparing patterns of molecular variance in the case of codominant microsatellite data (GenAlEx Manual and Appendix). The hierarchical distribution of genetic variation among populations /countries (*ie* within biotype) and within populations (among individuals) was calculated using 9 loci, 132 samples from seven populations for biotype B, and 11 loci, 213 samples from 9 populations for biotype Q (Table 2). AMOVA within Q biotype (Western and Eastern Mediterranean genetic groups) was done by subdividing the dataset accordingly. Tests of significance were performed using 9999 permutations.

**Table 2:** Analysis of molecular variance (AMOVA) results for biotype B and biotype Q populations.

	Source of variation	df^a^	SS	MS	Estimate of variance	% of total variation	*Φ*-Statistics
**Biotype B**	Among populations / countries (within B biotype)	6	209.094	34.849	1.714	27%	*Φ*_*PT*_ = 0.271*
	Among individuals (within populations)	125	575.315	4.603	4.603	73%	
	Total	131	784.409		6.317	100%	
**Biotype Q**	Among populations / countries (within Q biotype)	8	791.194	98.899	4.031	46%	*Φ*_*PT*_ = 0.458*
	Among individuals (within populations)	204	972.360	4.766	4.766	54%	
	Total	212	1763.554		8.798	100%	
**Biotype Q–Western Mediterranean**	Among populations / countries (within Q biotype West Med)	4	125.827	31.457	1.270	20%	*Φ*_*PT*_ = 0.198*
	Among individuals (within populations)	103	529.766	5.143	5.143	80%	
	Total	107	655.593		6.413	100%	
**Biotype Q–Eastern Mediterranean**	Among populations / countries (within Q biotype East Med)	3	230.158	76.719	2.794	39%	*Φ*_*PT*_ = 0.389*
	Among individuals (within populations)	101	442.594	4.382	4.382	61%	
	Total	104	672.752		7.177	100%	

Estimates of *Φ*_*PT*_ were calculated using 9 loci (excluding WF2C01, BEM6, BT-e49, BtIs1.2) on 132 samples from seven populations for biotype B and 11 loci (excluding BEM6, BT-b103) on 213 samples from 9 populations for biotype Q. Analyses within biotype Q were done on relevant subsets of the dataset, for Western and Eastern Mediterranean populations.

^a^df: degrees of freedom, SS: sum of squares, MS: mean squares

*significant at P<0.0001 (based on 9999 permutations)

## Results

### Basic population genetics statistics

Out of the 10,907 genotypes we attempted to obtain (13 loci x 839 samples), 1,829 failed to amplify consistently in certain populations (16.8% of dataset) with only 136 missing genotypes (1.2%) arising from ambiguous or non-specific PCR amplification. The complete dataset of 13 loci was used only for estimation of null alleles and the NJ tree, with other analyses done on reduced datasets, excluding loci deviating from HWE and missing data, as indicated in each case.

Tests for population differentiation showed that six sets of samples, each collected from neighboring locations, had non-significant population differentiation (*P* > 0.05 after Bonferroni correction) and were pooled together resulting in a total of 41 populations to be further analyzed (S1 Table).

High allelic richness was evident in all microsatellites (S2 Table), with 10 to 42 alleles per locus resulting in a total of 271 alleles across the 41 populations. When patterns of deviation were examined at the locus level, three loci deviated significantly from HWE in more than one population (S2 Table). No significant linkage disequilibrium was detected between loci.

**Fig 1:**
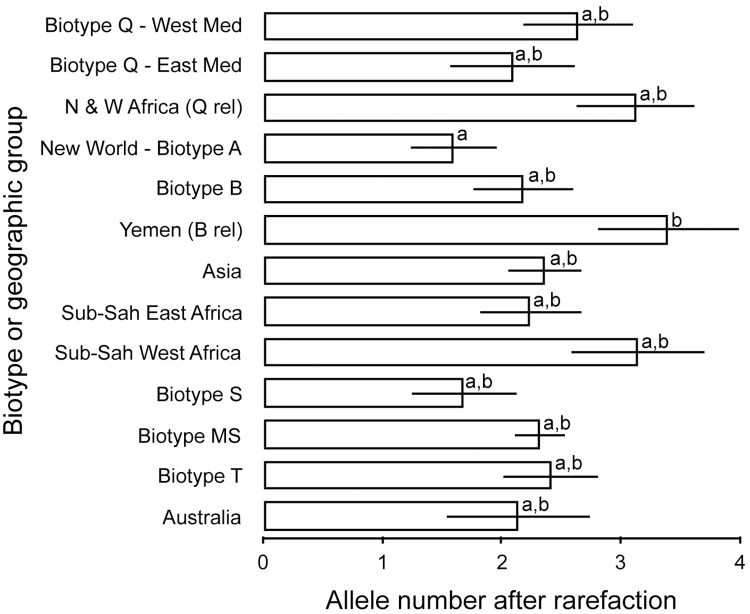
Allelic richness (±SE) (corrected for unequal sample size after rarefaction) in different biotypes and geographic groups. Different letters above the error bars denote statistically significant differences (*P* = 0,02).

The results indicated that different patterns of genetic diversity exist among biotypes and geographic groups. Fig 1 shows the number of alleles after rarefaction averaged across seven loci for each of the different biotypes or geographic groups. There were significant differences in allelic richness between populations (*F*_12,72_ = 2.26, *P* = 0.02). Populations from the New World (including biotype A) and biotype S had the lowest number of alleles, while the invasive biotypes B and Q (West and East Med refer to subclades Q1 and Q2 respectively as per Chu et al. [84]) had an intermediate number of alleles. The highest number of alleles was observed in the Yemen population, the presumed closest relative of biotype B represented in the dataset, and in populations from Sub-Saharan West and North and West Africa that is the presumed closest relatives of biotype Q sister clade represented herein (Fig 1, Table 3). However, the only statistically significant difference in allelic richness was found between the Yemen population and the New World (biotype A) populations (*P* = 0.04) (Fig 1).

**Table 3:** Population specific statistics over 13 microsatellite loci.

Population name^a^	Na^b^	*H*_E_^c^	*H*_O_^d^	*F*^e^
Arizona A	1.462 (0.573)	0.079 (0.057)	0.064 (0.058)	0.305 (0.167)
Arizona B	4.923 (0.755)	0.436 (0.089)	0.408 (0.093)	0.101 (0.059)
Australia	1.462 (0.369)	0.137 (0.075)	0.154 (0.087)	-0.150 (0.116)
Burkina Faso 3, 4, 6	8.846 (1.754)	0.573 (0.075)	0.453 (0.084)	0.204 (0.098)
Cameroon 2, 14	4.538 (0.685)	0.559 (0.047)	0.479 (0.086)	0.175 (0.123)
CanIsl-1, 3, 4, 6 (Canary Islands)	4.769 (1.063)	0.456 (0.078)	0.439 (0.080)	0.067 (0.101)
China	2.692 (0.414)	0.308 (0.072)	0.280 (0.076)	0.101 (0.116)
China-Hainan	1.077 (0.309)	0.093 (0.059)	0.077 (0.049)	0.069 (0.069)
Cyprus 2006	5.462 (1.004)	0.446 (0.096)	0.414 (0.088)	0.052 (0.041)
Cyprus 2008	4.385 (1.071)	0.348 (0.083)	0.356 (0.084)	-0.009 (0.046)
Cyp (Cyprus-Ork)	1.077 (0.288)	0.173 (0.066)	0.154 (0.067)	0.040 (0.162)
Egypt	6.385 (1.135)	0.515 (0.070)	0.441 (0.075)	0.177 (0.090)
France	3.692 (0.429)	0.453 (0.059)	0.480 (0.071)	0.000 (0.089)
Greece-1, 3	4.615 (0.888)	0.439 (0.090)	0.361 (0.079)	0.137 (0.066)
Greece-2	3.769 (0.907)	0.427 (0.058)	0.276 (0.060)	0.329 (0.128)
Guatemala	0.923 (0.288)	0.078 (0.054)	0.038 (0.038)	0.379 (0.243)
India	2.077 (0.487)	0.240 (0.073)	0.233 (0.081)	0.127 (0.130)
Israel Q	3.000 (0.494)	0.357 (0.084)	0.351 (0.088)	0.016 (0.057)
Israel-3, 4 B	3.615 (0.549)	0.462 (0.080)	0.568 (0.102)	-0.205 (0.053)
Italy T	2.154 (0.576)	0.297 (0.084)	0.279 (0.081)	0.045 (0.057)
Ivory Coast	3.769 (0.590)	0.453 (0.080)	0.345 (0.087)	0.268 (0.117)
Jatropha-Puerto Rico	0.846 (0.222)	0.048 (0.048)	0.038 (0.038)	0.200 (0.064)
Mexico	1.615 (0.432)	0.167 (0.071)	0.093 (0.051)	0.370 (0.118)
Mexico-Culiacan	1.308 (0.511)	0.077 (0.052)	0.074 (0.064)	0.216 (0.190)
Moorea-French Polynesia	0.538 (0.215)	0.038 (0.026)	0.044 (0.030)	-0.167 (0.000)
Morocco	3.538 (0.573)	0.431 (0.080)	0.378 (0.086)	0.097 (0.102)
Mozambique	2.538 (0.867)	0.294 (0.095)	0.118 (0.051)	0.610 (0.079)
Pakistan K	2.538 (0.647)	0.275 (0.078)	0.241 (0.070)	0.120 (0.056)
Panama	3.154 (0.390)	0.461 (0.068)	0.459 (0.077)	-0.006 (0.076)
Reunion-MS	2.154 (0.296)	0.275 (0.063)	0.117 (0.039)	0.552 (0.099)
Reunion-MS-2009	3.308 (0.499)	0.427 (0.066)	0.477 (0.090)	-0.114 (0.108)
Riverside A	1.308 (0.485)	0.080 (0.054)	0.049 (0.041)	0.403 (0.154)
South Africa	2.462 (0.627)	0.264 (0.080)	0.157 (0.064)	0.325 (0.120)
Spain S	1.769 (0.568)	0.132 (0.067)	0.071 (0.034)	0.333 (0.078)
Spain	4.538 (0.985)	0.416 (0.066)	0.379 (0.077)	0.065 (0.093)
Sudan	3.308 (0.737)	0.424 (0.093)	0.359 (0.092)	0.151 (0.098)
Sudan Q-like	2.692 (0.308)	0.383 (0.060)	0.431 (0.075)	-0.092 (0.068)
Turkey M	2.615 (0.417)	0.302 (0.079)	0.253 (0.074)	0.115 (0.109)
Uganda-cassava	1.615 (0.500)	0.226 (0.084)	0.152 (0.081)	0.388 (0.160)
Uganda-sweetpotato	3.538 (1.084)	0.324 (0.097)	0.229 (0.083)	0.290 (0.102)
Yemen 1, 2 –B_2_	5.385 (0.656)	0.624 (0.060)	0.532 (0.069)	0.105 (0.104)

^a^Samples collected from neighboring locations, which showed non-significant differentiation (exact tests of population differentiation) were pooled together, as shown in S1 Table.

^b^Mean number of different alleles over loci (±SE)

^c,d^Mean expected (*H*_E_) and mean observed (*H*_O_) heterozygosities over loci (±SE)

^e^Mean fixation index (*F*) over loci (±SE)

**Fig 2:**
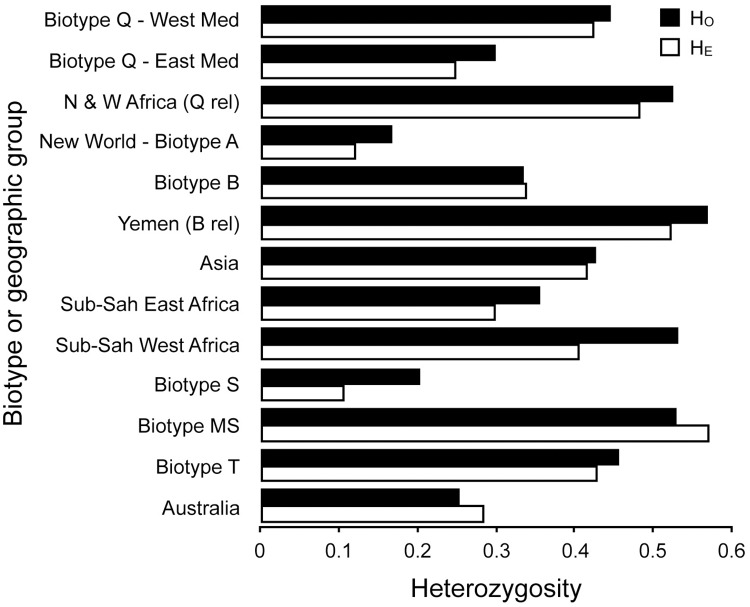
Observed (*H*o) and expected (*H*e) heterozygosity in different biotypes and geographic groups.

Estimates of heterozygosity showed similar patterns as allelic richness: the introduced to Spain, S biotype, and New World populations had the lowest heterozygosity; the invasive biotypes B and Q had moderate to high heterozygosity, while the highest levels were observed in their close relatives extant in Yemen and the North and West African lineages, and for another related biotype referred to as Ms from Reunion Island (Fig 2, Table 3), a haplotype first identified in Uganda (Ug) and initially referred to as the Ug-non B [85].

### Null alleles

**Fig 3:**
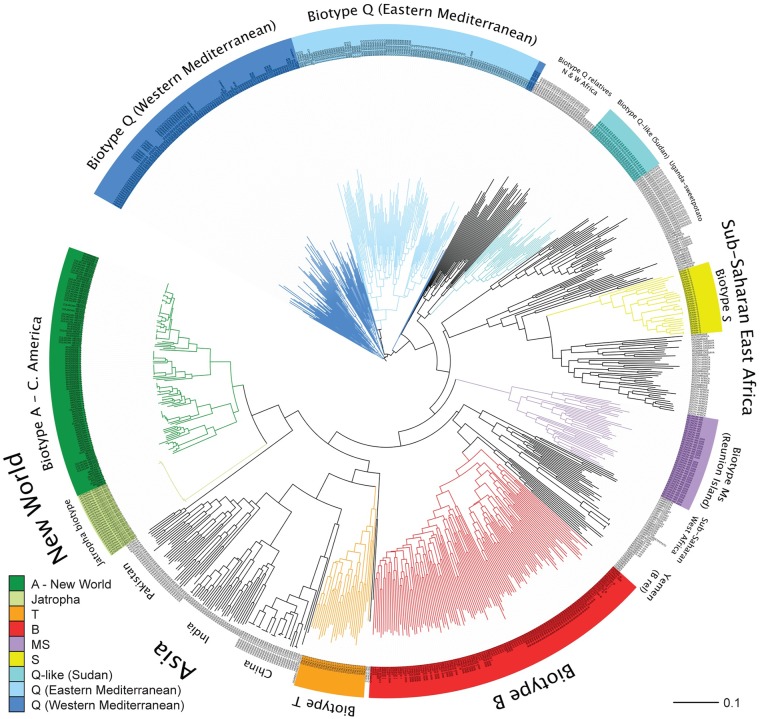
Unrooted NJ tree based on Cavalli-Sforza & Edwards chord distance (*D*_*C*_) among individuals. The tree was constructed using the full dataset of 839 whiteflies from 50 collections, genotyped at all 13 microsatellite loci used in this study. Colored clades and branches represent biotypes previously characterized based on mtCOI data and biological/ ecological information. Other groupings are named according to geographic structuring of populations.

Although analysis using FreeNa [66] showed that null alleles were present in the dataset (S3 Table), estimates from the corrected and non-corrected dataset were very similar. In the case of *F*_ST_, the global estimate across all loci and populations was 0.54 (95% CI: 0.46–0.62) before correction for null alleles and 0.53 (0.45–0.61) after correction. Per locus estimates of *F*_ST_ with and without the correction were also very similar, with only six out of 13 loci having slightly overestimated values before the correction and the largest difference between corrected—uncorrected dataset at a locus being 0.03 (locus WF1B06). The NJ trees built based on *D*_*C*_ calculated with both the corrected and uncorrected for null alleles dataset gave identical topologies (not shown), that were consistent with the groupings recovered from individual NJ trees (Fig 3).

### Individual-based analyses

#### Neighbor Joining tree

The NJ tree based on individuals gave a clear picture of the genetic structure associated with the biotype/haplotype, or geographic origin, with the exception of the known introduced, invasive populations (Fig 3). A total of 18 groups, including seven established biotypes, showed clear genetic structure, with individuals within each group being more related to each other than to individuals from any other group.

Within the Q biotype clade there was a obvious split into two sister genetic groups originating from the eastern Mediterranean (including the Israel-Q) and those sampled from the western Mediterranean (including the Spanish-Q), a genetic split also evident based on the mtCOI data [84]. The Eastern Mediterranean Q cluster was more structured than the West Mediterranean Q and comprised well-differentiated populations that included those from Cyprus, Greece, Israel, and Turkey, whereas, there was evidence of more gene flow within the Western Mediterranean group that included those from Spain, Morocco, France, Canary Islands, and a population introduced to China.

The B biotype formed a monophyletic group with evidently high migration among its five sister populations, with the exception of the Arizona B population that was well differentiated from the rest. Other biotypes that formed monophyletic groups in the tree were biotype S [86] (collected in Spain but now known to be extant in West Africa on cassava and probably other hosts), biotype Ms from Reunion Island, biotype T (Italy) [87, 88] and the Jatropha -PR biotype (Caribbean) [18, 89] whereas populations of the biotype A (Arizona A and Riverside A) [85, 89, 90] were not differentiated from the other New World populations collected in Central America.

Also, there were examples of individuals that did not belong to any previously recognized biotype, but that nevertheless formed clear and well-differentiated groups. Examples were haplotypes from Burkina Faso (Q-like relative), Sudan and Sudan Q-like, Moorea-FP (sister clade to Sub-Saharan, West African clade), Yemen (B biotype relative), and samples from China-Hainan, India, Pakistan (Asia I and II) and the Uganda-sweetpotato population. Haplotypes collected from cassava in Mozambique and Uganda and haplotypes from South Africa were not well-differentiated from one another, but formed one large clade, herein referred to as Sub-Saharan East Africa [24], with the S biotype (found initially in Spain but later in West Africa where it is thought to have originated) nested within it. A similar pattern, with unclear differentiation, was observed among individuals from Cameroon and Ivory Coast (Sub-Saharan, West African clade).

When within population patterns were examined, we observed high diversity among individuals within populations of biotype B despite the lack of structure among them. In contrast, the opposite pattern was observed for the monophagous Jatropha-PR biotype in that the 28 individuals we examined forming a distinct clade, and individuals were nearly genetically identical to each other.

An analysis that used a dataset of eight loci gave a tree with much less resolution within the clades containing the B and Q biotypes and their closest relatives, and between those and their closest relatives from Yemen and North and West Africa, respectively (since those excluded five loci amplified mostly in these populations). Otherwise, the analysis with eight loci did not affect the overall tree topology and resulted in the same groupings in other biotypes and geographic regions (results not shown).

#### Principal Coordinates Analysis

**Fig 4:**
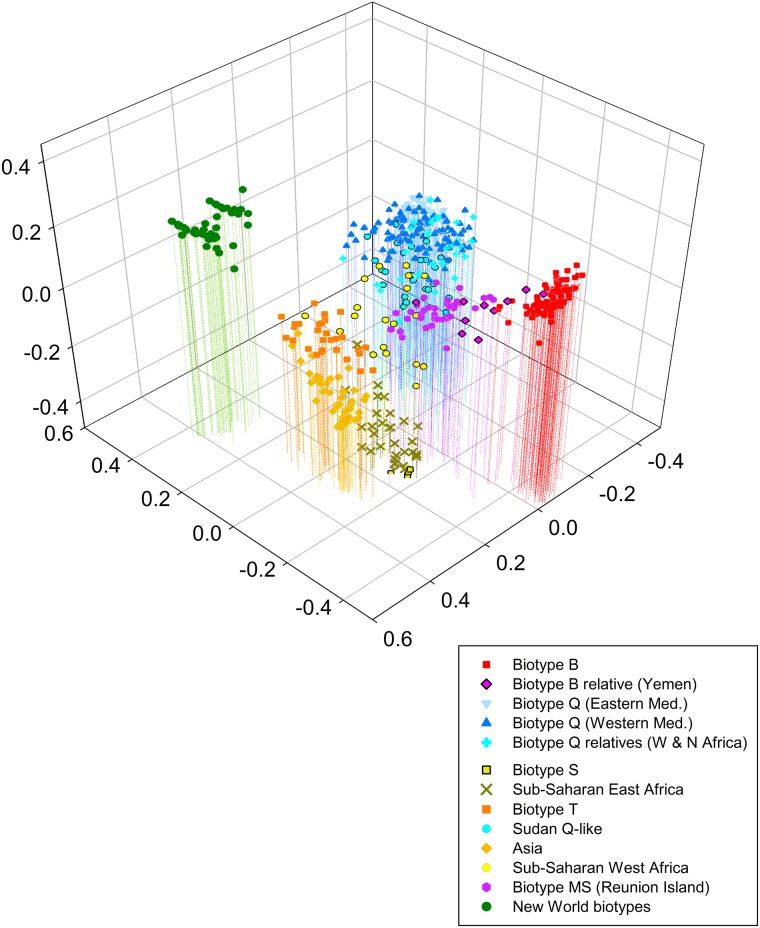
Three-dimensional plot of a Principal Coordinates Analysis based on a genetic distance matrix calculated from individual multilocus microsatellite genotypes. The PCA was produced using eight loci (WF1B11, BEM6, BEM15, BEM31, BT-b103, BT-e49, MS145, MS177) and a dataset of 712 individuals from 42 populations. Data from individuals and from populations (denoted in S1 Table with *) that had many missing data and added little value to the PCA was excluded from this analysis. Individuals are color-coded according to biotype or geographic group they belong to based on results from NJ tree with corresponding colors.

The 8-locus dataset used for the PCA had very little missing data (out of 5,696 possible genotypes only 82 or 1.4% failed to amplify). The PCA showed that the first three components explained a cumulative 72.3% of the total variance (Fig 4) of the data. Overall the PCA gave similar major patterns as the individual-based NJ tree (Fig 3). There were 7 clear clusters that corresponded to individuals from the New World, Asia, Sub-Saharan East Africa and biotypes S, T, Ms (Reunion), Uganda non-B [85, 91, 92], B, and Q. Within the biotype Q sister clade there was a split with some overlap between the Eastern and Western Mediterranean at the level of the second PCA axis, was visible when the axes were rotated. The Yemen individuals were loosely clustered between the B and Ms biotypes. A similar pattern was observed for individuals from the Q-relatives from West and North Africa (Burkina Faso, Sudan) and the Sudan-Q-like haplotype, which were collectively scattered between African populations and the biotype Q clade. Likewise, individuals from Cameroon and Ivory Coast (Sub-Saharan West Africa) did not form a cluster, but were scattered and mostly overlapped with the Q-relatives from West and North Africa. Overall, this analysis showed that the most differentiated cluster with the largest genetic distance from all others, at all levels of the PCA, was the New World cluster. The B and Q biotype sister clade groups, and the major Asian cluster were also very distinct, occupying the furthermost positions of the PCA axes.

#### Bayesian clustering analysis to assess worldwide population structure

**Fig 5:**
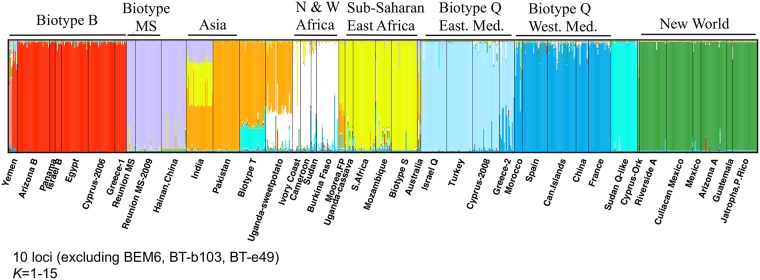
Bayesian clustering analysis results of worldwide multilocus genotypes of *B*. *tabaci* performed in Structure. Individuals are arranged on the x-axis, each represented by a thin vertical line and partitioned into each of 9 inferred clusters (*K*) with their estimated membership fractions on the y-axis. Labels below the plot represent the sampled populations and above the plot the biotypes or geographic groups. Clusters are colored according to groupings identified in other analyses (PCA, NJ tree). The number of *K* specified and the loci used are indicated below the plot.

The initial results from the clustering analysis that included all individuals in Structure revealed the presence of 9 genetic groups that in general corresponded to biotype designations (Fig 5). However some populations, such as those from India, the T biotype, and the Uganda-sweet potato had mixed estimated membership coefficients, and unclear assignments into clusters between replicate runs. In addition, in the multiple runs attempted, the correct value of *K* varied, with the Evanno et al. [77] procedure indicating the presence of high peaks in Δ*K* at *K* = 4, and 9 with smaller peaks at K = 11 and 14 (S1 Fig). Here we chose to present *K* = 9 because when the posterior probabilities were plotted [Pr (*K*)] against *K*, Pr (*K*) plateaued at *K* = 9 (S2 Fig). Plots showing clustering results at *K* = 8, 10 and 11 are also presented as supporting information (S3, S4 and S5 Figs).

**Fig 6:**
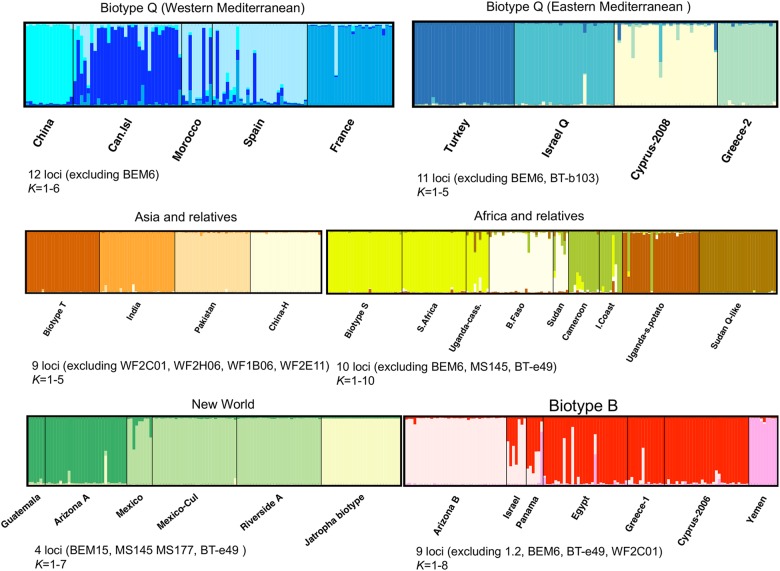
Bayesian clustering analysis results of multilocus genotypes of *B*. *tabaci* from different biotypes or geographic groups performed in Structure (sub-structure analysis). Individuals are arranged on the x-axis, each represented by a thin vertical line and partitioned into each of the inferred clusters (*K*) with their estimated membership fractions on the y-axis. Labels below the plot represent the sampled populations and above the plot the biotypes or geographic groups. The number of *K* specified and the loci used in each run are indicated below each plot.

After subdividing the dataset by biotype or by geographic group level (based on results from the NJ tree and *a priori* knowledge of population affiliations with biotypes), it was possible to identify a number of clusters *K* by examining both the posterior probabilities of the data against *K* and the Δ*K* estimator. Results from these sub-structure runs revealed some interesting patterns (Fig 6). In the Western Mediterranean the Q biotype and related haplotypes split into four clusters, consisting of China, France, Morocco and Spain, and the Canary Islands, with the last two clusters seemingly sharing migrants. France and the population introduced into China [91, 92] were well differentiated, while individuals from Canary Islands, Morocco and Spain shared a fraction of their genotypes with France and China. A different picture was observed in the Eastern Mediterranean Q biotype plot, for which a clear genetic structure was observed among all four populations. Thus, there seems to be very little gene flow between the two Q biotype sister groups (Eastern and Western) (Fig 5), with mostly the Greek populations from the Eastern Mediterranean sharing some ancestry with the Western Mediterranean cluster. The result was similar and clear when all Q biotype-like populations (Eastern and Western) were included in a single STRUCTURE run (S6 Fig).

There was a similar pattern for the Asian populations and their relative, the T biotype from Italy, with all populations well differentiated and no evidence of gene flow among them.

In the analysis of African populations, we excluded Mozambique (which in other analyses clustered with Sub-Saharan, East African populations) in order to use as many loci as possible since this population deviated from HWE in an additional two loci. Biotype S, a South African haplotype, and some Uganda cassava-colonizers formed a single cluster, populations from Burkina Faso and Sudan formed another (by mtCOI these group with others in the Q-like clades, sisters to B-like clades), while Cameroon and Ivory Coast haplotypes grouped together as a separate cluster. The Ugandan-sweetpotato (perhaps not *B*. *tabaci*, or a divergent lineage) and Sudan Q-like populations were genetically distinct from all other clusters. Despite the well-defined structure in this plot, some admixture and migration was detected between the Western and Eastern African clusters.

Genetic structure was also observed within the New World, with the Jatropha-PR population forming a distinct cluster, and a clear differentiation between the Arizona A biotype (e.g. prototype) and the Riverside A haplotype, with the former being most genetically similar to the Guatemalan population (Caribbean-Central America), and the second with the populations from western Mexico.

Moderate structure was observed in the B biotype-closest relatives analysis. The Arizona B biotype was genetically different from the Egypt, Greece-1 and Cyprus-2006 populations, which collectively formed a single cluster, with only fractions of individuals’ genotypes sharing ancestry with Arizona B. The B biotype sister clade relatives from Israel and Panama (exotic introduction there) were admixed between the Arizona B (prototype) cluster and the B-like cluster consisting of Egypt, Greece, and Cyprus collections. Finally, the population from Yemen was genetically distinct from all B biotype populations.

The results of the analysis using Baps identified 28 genetic clusters worldwide, was generally in agreement with the Structure results when examined at the biotype/geographic region level (results not shown). Because in Baps we included all populations, this analysis also identified as genetically distinct the samples from Reunion Ms, Reunion Ms-2009, Cyprus-Ork, Moorea-FP, and Australia, which were excluded from the sub-structure analysis carried out using Structure because they were not associated with a larger geographic group.

Genetic differentiation within the two invasive biotypes B and Q based on the *Φ*_*PT*_ estimate *via* AMOVA indicated that most of the genetic variance in both biotypes was significantly partitioned within populations with less variance among populations (P<0.0001). This was particularly the case for B biotype where 73% of variance was within populations (*Φ*_*PT*_ = 0.271; P<0.0001) and for Western Mediterranean Q biotype with 80% of variance partitioned within populations (*Φ*_*PT*_ = 0.198; P<0.0001) (Table 2).

## Discussion

### Genetic diversity and population differentiation at the worldwide level

Estimates of genetic diversity, measured as allelic richness and heterozygosity showed variable patterns for different biotypes. The highest levels of allelic richness and heterozygosity were found in the populations from Sudan and Burkina Faso, and Yemen (Table 3, Figs 1 and 2) that are the closest relatives of biotypes Q (Spanish Q) and B (Arizona B) and as has been found in previous studies based on variation in mtCOI [24, 30]. High levels of heterozygosity were also observed for the Ms biotype from Reunion Island, which also was shown to be a relative of B biotype in this study (Fig 4) and elsewhere [24, 30], and as a sister clade of biotypes B and Q in the mtCOI phylogeny [93, 94]. Overall, results show that populations from eastern Africa and the Middle East have the highest microsatellite diversity within and among populations, suggesting that they represent old lineages that gave rise to the extant invasive B and Q biotypes (at ~16–26%), which group within the major Mediterranean-North African-Middle East clade [24], thereby, potentially pinpointing to this vicinity the origins and diversification of the *B*. *tabaci* invasive haplotypes that group within the B and Q sister clades.

Levels of allelic richness and heterozygosity in the populations examined may have been influenced by two additional factors: the effects of inbreeding in samples obtained from lab colonies, and because microsatellite loci can be more variable in biotypes from which they were isolated [95, 96]. In addressing the first factor, we observed low diversity in some samples that originated from sustained lab colonies: in the prototype Arizona biotype A collected from cotton fields in Phoenix, AZ, in a geographically proximal relative, the Riverside A (from Imperial Valley, CA), and in the possibly monophagous S biotype that was collected in Spain but also extant in western Sub-Saharan Africa) and maintained in a laboratory culture. However, field collected A-like populations from the Sonoran Desert habitat of the northwestern states in Mexico Sonora and Sinaloa (located immediately south of Arizona, USA from where the Arizona-A and Riverside-A are indigenous) had similar estimates, as did the Arizona A biotype, maintained as a lab colony since 1981. Similarly and perhaps surprisingly as well, samples from other lab colonies (Arizona-B, Israel-Q-like, and Italy T) had moderate to high allelic richness and heterozygosity, similar to field haplotype or biotype populations from the same region. Thus, although laboratory rearing of some populations may play a role in the estimates obtained herein, it is likely that the demographic histories of these populations (such as ancestral population bottlenecks) account mostly for the pattern we observed. For example, the low genetic diversity observed in New World populations (lab or field collected) is consistent with data from the mtCOI phylogeny, which shows strikingly less within-clade divergence (~4–8%) in New World compared to Old World clades (14–26%) [24, 97]. The species-specific variability of microsatellites that might have influenced patterns of allelic richness and heterozygosity in the samples analyzed here was compensated in this study because the loci that were used represented 4 different genetic groups/biotypes (Asia, Australia, Arizona-B biotype, Spanish-Q biotype), thereby minimizing this effect in our results and conclusions.

The microsatellite analysis of *B*. *tabaci* populations revealed large genetic distances at the worldwide level, and suggests that this taxon consists of very divergent cryptic lineages that represent geologically old entities (see Results and Figs 3, 4 and 5). These findings are in line with previous studies of cryptic species that suggested divergence dating back to millions of years ago, despite morphological conservatism [10, 14, 15]. The results based on nuclear microsatellites show that these lineages correspond to described and well-studied “biotypes” that have been characterized on the basis of allozyme differences, phylogenetic analysis of the mtCOI gene, and biological/ ecological assays such as host-feeding, virus transmission and crossing experiments [18–20, 22–24, 30]. Furthermore, other distinct genetic groups identified in these analyses have a geographic basis, with the exception of ‘known’, recently invasive and introduced haplotypes, and are in general agreement with the mtCOI phylogeny of the sibsp. group (see S1 Table for population affiliations with mtCO1 clades). The PCA showed that the most divergent lineage of those we examined is that consisting of New World populations, followed by biotypes B, Q, and the Asian populations, with no evidence for gene flow among them (Fig 4). Although the results suggest older divergence among these groups compared to any others, divergence date estimates would require molecular dating analysis of phylogenetic clades since microsatellites are unsuitable for inferring deep phylogenetic relationships. Indeed, such a study, recently undertaken using molecular dating of the mtCOI gene for *B*. *tabaci*, estimated that New World and Asian lineages had some of the oldest divergence times (estimated at 44 MYA), predating the divergence of most other clades/genetic lineages within the species group, in line with our results [22, 27].

Another indication of the extreme divergence in *B*. *tabaci* emerging from our results is that of the 13 loci we used, seven failed to amplify in samples from some populations. Non-amplification of microsatellite loci by PCR is most often caused by poor primer specificity due to mutations in regions flanking the microsatellite repeat sequence, resulting to what is known as “null alleles” [98, 99]. Although null alleles can occur at a low frequency at the species level, this frequency increases with increasing phylogenetic distance at the genus level [61]. This *a priori* observation is also in line with conclusions herein, because microsatellite loci that failed to amplify did so systematically within a biotype or geographic group, consistent with the presumed genetic relationships among populations, with non-amplifying loci occurring mostly in individuals of the most divergent lineages (*e*.*g*. New World) (Fig 4). Despite the null alleles identified, the genetic differentiation estimates (*F*_ST_) using corrected and uncorrected datasets were very similar. In addition, the NJ trees constructed based on *D*_*c*_ corrected for null alleles gave the same tree topologies as uncorrected trees, suggesting that null alleles had minimal impact in the analyses and did not affect the conclusions.

### The invasive biotypes (B and Q)

The most well-known biotypes in the *B*. *tabaci* sibsp. group currently are the invasive haplotypes that group in the clusters contained within the main B and Q biotype ‘sister’ clades. Some B-like haplotypes have expanded their geographic range to a worldwide scale in the past 30 years, while the Q-like invasive types have done so more recently [100–105]. Many other biotypes and genetically distinct haplotypes around the world, such as the cassava and sweetpotato populations found in Africa [85, 106] are pests and plant virus vectors of great local and regional importance in sub-Saharan Africa. Thus far some of these cassava types have remained restricted to their presumed endemic locales, while certain of them have become invasive and are now widely distributed (e.g. since the 1990’) in sub-Saharan Africa where they transmit a suite of highly damaging begomoviruses to cassava [106].

The factors that have favored the worldwide expansion of the B and Q biotypes *per se* have yet to be determined; but the most likely explanation is that the direction of global trade of infested plant products has facilitated these invasions into locations having both a similar climate and susceptible plant host species. From this and previous studies [18, 19, 30], it does not seem that other non-B-like or non-Q-like populations have expanded their range worldwide and become invasive, or at least to a similarly detectable magnitude. It is likely therefore that certain inherent characteristics of B- and Q-like haplotypes (notable in the prototype biotypes) have also contributed to successful invasions and displacements of local biotypes, for example, the A biotype was rapidly displaced by the B in the US and in other locales in the American Tropics within a very short timeframe [18, 89]. Further, the AZ-A, AZ-B, and Q1 biotypes are known to exhibit differential resistance to certain insecticides [91, 107–110]. The widespread invasion of B and Q biotypes into new areas [100, 101] where local biotypes are still susceptible to certain types of insecticides could readily provide the invaders a competitive advantage and with time their populations built up, driving local biotypes to extinction if they cannot interbreed (compatibly) with them.

Here, results indicated that the invasive biotypes B and Q did not have lower genetic diversity when compared to that of all other biotypes (Figs 1 and 2), which would be an expected pattern in invasive populations under bottlenecks from multiple founder effects, and directional selection, for example, from intensive agriculture (e.g. insecticide applications). In fact, estimates of allelic richness and heterozygosity were high in most populations of biotypes B and Q, indicating high levels of genetic diversity [50, 51]. Additionally, despite the relatively low structure observed in the clades containing the B biotype (and relatives), and the Western Mediterranean Spanish Q and Q-like relatives (Figs 3 and 6), we found substantial variation among individuals, especially within (Western) Q relatives, with all multilocus genotypes being unique and most of the variance partitioned among individuals. The moderate diversity in the invasive B biotype indicates a large effective population size and an ancestral lineage, and suggests that the presence of ancestral variation has likely resisted the homogenizing effects of human-mediated gene flow among populations. The fact that B biotype and Western Mediterranean Q1 and Q-like relatives showed less structure and more percentage of variance partitioned among individuals rather than among populations suggests extended gene flow and substantial differentiation at the population level, likely maintained by continuous invasion events across countries and regions. This is the opposite of what we observed, for example, in the non-agriculturally important, monophagous Jatropha-PR biotype where almost all individuals had identical genotypes and no differentiation (Fig 3), possibly a result of its host-specialization on the genus, *Jatropha* and closely related euphorbiaceous species in the Caribbean Islands. So, despite the genetic bottlenecks induced by founder effects in these invasive biotypes, genetic diversity remains high, possibly either due to persistent ancestral variation or due to continuous and repeated migrations from multiple diverse source populations from different regions, or both. This latter phenomenon has been observed in other organisms [111, 112], and so in the case of *B*. *tabaci* it would be facilitated by human-mediated transport on whitefly-infested plants around the world, from multiple source populations originating in relatively close proximity to one another, the upper northwestern portion of Africa and the Middle East-Arabian Peninsula.

Several important patterns of population differentiation were evident within invasive types of B and Q. Within the Q biotype there exists a large split in the Western and Eastern Mediterranean lineages that correspond to what are known as the Spanish-Q (Western) and the Israel-Q (Eastern) [24, 113, 114] or Q1 and Q2 subclades based on mtCOI haplotypes [46, 50, 51, 84, 92]. While they are both placed in the so-named Q clade (using the Spanish Q as the prototype), they clearly represent distinct and divergent lineages with little gene flow between them, a puzzling result, despite their occurrence in a zone where the Mediterranean Sea connects Spain and nearby islands, with the Middle East. Furthermore, there seems to be high genetic structure with limited gene flow within the Eastern Mediterranean-Q populations, but substantial gene flow among most of the populations examined from the Western Mediterranean Q-clade, with most of the genetic variance partitioned among individuals rather than among populations. High levels of differentiation within Western Q1 populations were also reported in a study that examined samples from a large number of populations across the Mediterranean [50]. The same study also found that the two Q lineages coexist in Spain and France either separately or in sympatry with evidence for asymmetric gene flow between them. Interestingly, the two Q biotype lineages also seem to differ in endosymbiont composition [38, 50–52, 115]. It will be interesting to determine which of the two Q-like lineages has more potential for invasiveness outside their native Mediterranean range. In this study an invasive Q haplotype that invaded China grouped within the Q1 western lineage. In Italy a study showed that the Q2 mitochondrial type (Eastern Med) has invaded, possibly favored by the agroecological conditions of southern Italy, by the female-biased sex ratio or perhaps by endosymbionts acting as sex-ratio manipulators [115]. Results from another study suggested that at least the US invasion of the Q biotype stemmed from both the Western Q1 and the Eastern Q2 lineages [54], a result that can be supported based on knowledge of routes of ornamental and perhaps other kinds of traded plants that are hosts of *B*. *tabaci*. Interestingly, an invasive Q1 population reported in China has displaced the previously invasive B biotype in at least certain locations, particularly in certain vegetable crops [92]. To what extent the two lineages have biological and ecological differences remains to be further studied [116]; they originate from proximal geographies and apparently both possess high inherent, differential, resistance to presently used insecticides in agricultural production [50, 113, 117], and which could logically have been a driving force behind their establishment outside their indigenous zones that contain highly managed agricultural systems.

Within the B biotype/haplotype collections examined there was high gene flow among populations except for Arizona B, which, perhaps surprisingly, was well differentiated from the others. The Arizona B (prototype B) originated from poinsettia plants that were subsequently reared as a lab colony in Arizona (JK Brown laboratory) when it was first recognized as an invasive *B*. *tabaci* in the US during 1987–88, based on a unique, homogeneous esterase pattern. This was followed by a number of apparently parallel introductions on ornamental plants and by its rapid spread throughout the Americas during the 1990’s-onward (colonizing poinsettia plants), and subsequently to Japan [100], China (see references in [92]) and elsewhere. It was the reference population for the esterase-based characterization of the local Arizona (prototype A) and exotic B populations along with the Arizona prototype B from poinsettia [90], and a number of additional geographical variants (C through T) [89]. The differentiation observed for subsequent invasive B haplotype populations may simply be due to temporal changes in allele frequencies since 1990; however we did not see much differentiation in populations from Egypt and Cyprus, which were sampled with a gap of five years. The founding effect in the Arizona B prototype colony (from imported poinsettia plants) may be associated with this differentiation, because by laboratory-based isolation only a subset of the introduced population was selected and bred by serial transfer over multiple generations. The shared ancestry of Arizona B with the population from Israel suggests that the B biotype introductions in the USA may have originated from this area of the Mediterranean owing to trade; indeed the esterase pattern is identical to that reported for an indigenous Israeli population by Wool et al. [118], a location among others where the use of new synthetic pyrethroids had been recently instituted, as was also the case in the US.

The exact origins of the invasive B-like and Q-like biotypes have yet to be determined, but it has been suggested that the so-called Spanish Q biotype originates in southern Spain and has relatives in Northwest Africa and elsewhere in the Mediterranean and Middle East, while the B biotype diversified in the eastern African Sahel-Middle East (see references and data in [24]). Consistent with these hypotheses are our findings of genetic similarities of biotype Q with North and West African populations and of biotype B with the Yemeni population. Understanding the origins of these biotypes and timing of divergence from their closest relatives are critical for identifying attributes that facilitated their invasiveness and their high pest status.

### Other notable biotypes

The *B*. *tabaci* sibsp. group represents an excellent system for studies of cryptic speciation where evolution has favored a wide array of genetically, ecologically, and biologically diverse lineages around the world. One application is in understanding differences between invasive and non-invasive biotypes, and how these have shaped their evolution and adaptation.

The T biotype was first identified in Italy in 2003 colonizing only *Euphorbia characias* in a high altitude area [87, 88, 119]. The population examined here from Puglia, Italy of this biotype appears to be a distinct *B*. *tabaci* lineage, but is clearly genetically related to the Asian populations, which agrees with existing literature based on mtCOI [19, 22, 24, 120]. The T biotype likely represents an ancestral introduction of populations into the Mediterranean from Asia, or remnant populations of a wider historical distribution of Asian lineages whose range later contracted. An extensive sampling of high altitude areas in the Mediterranean, away from agricultural areas and greenhouse establishments where biotypes B and Q are likely to be found could provide more information to test these hypotheses. Indeed in one of our sampling efforts in the island of Cyprus in 2008, we found 2 individuals in a mountainous area far from agricultural fields, which in our microsatellite tree formed a clade sister to the Asian clade that includes the T biotype. Haplotypes of this new variant, which we call Cyp were also shown to form a clade sister to T biotype in a mtCOI phylogeny, with ~8% sequence divergence between the two (authors, preliminary data). Further collecting in such areas to obtain individuals for biological and ecological assays would determine whether the population we sampled these whiteflies from represents a distinct biotype, relative to the Asian populations, like the T biotype. Another study [121] found another genetic variant in Southern Italy collected from *Rubus ulmifolius* and grapevine, referred to as Ru, whose haplotypes formed a sister clade to the Asian and Australian groups, and the clade consisting of T biotype with which they shared 10.7% pairwise genetic distance. Clearly the T biotype and its relative genetic variants in the Mediterranean make a very interesting case that should be further explored. With appropriate genetic analysis of these and other historical samples it would be possible to date their divergence from other close relatives and to determine whether they represent introductions or remnants of ancestral widespread distribution in the Mediterranean region.

The S biotype, which was first described from the weed *Ipomoea indica* in Spain in 1995 [86, 122] is most likely a relative of African origin since similar populations are extant there (JK Brown, unpublished results) and so it has probably been previously introduced into Spain but did not become widespread in the region [85, 123]. The microsatellite analysis here showed that this population forms a clade nested within the Sub-Saharan East African clade which includes Mozambique and cassava-associated populations from Uganda, and South Africa, consistent with previous studies based on mtCOI [93, 120], ITS I sequences [124] as well as AFLP analysis [123]. Genetic diversity estimates indicated that this population had lower allelic richness compared to other African populations, which suggests that a subset of the source population was introduced and survived in the sampled area, likely undergoing a genetic bottleneck. Although this biotype had only been previously reported in Spain outside its African native range, a recent study also reported its presence in Southern France [125]. In that study, it was shown that microsatellite genotypes from individuals originating from a French Q biotype (Western Mediterranean) sample were not clearly assigned to the biotype S population, but seemed to have admixed ancestry between the Q and S biotypes. This suggests sex-biased admixture, with females from the S biotype retaining their maternal mtDNA but exchanging genes with males from the Q biotype. A parallel result, albeit, less well characterized, has been reported for two populations in Uganda that colonize cassava, although one is thought to perhaps be polyphagous, and the other monophagous on cassava, possibly making it less fit [106]. Although speculative, perhaps this admixture (hybridization) was induced by the maternally inherited *Wolbachia* or *Cardinium* endosymbionts, known to at times drive uni-directional gene flow between infected and uninfected populations [126–130]. The S biotype collections analyzed so far suggest that this haplotype occurs in small populations in the Western Mediterranean, and in some cases in co-existence with the Q biotype. In Africa, the S biotype also occurs intermixed with haplotypes associated with cassava and vegetable growing regions, though it is not clear whether there it is monophagous (or nearly so). This is because it has been found only in small numbers and on varying host species, but whether those species are reproductive and/or feeding hosts is not known. A question that arises is why has this or many other biotypes not become invasive after their introduction in their area or other very different locales, as has the biotype B? Possibly, the competition with biotype Q and/or lower levels of insecticide resistance and/or host-adaptation do not allow these populations to built-up and expand. It is also possible that like its cassava-restricted relatives in Africa [124], biotype S cannot easily adapt to other hosts and become an invasive pest in the region. The distribution of the S biotype in the Mediterranean and its ecological and genetic interactions with the other prevalent biotypes in the area makes a very interesting case to be studied extensively in the future.

### Whitefly population structure inferred using nuclear microsatellites compared to a mitochondrial DNA marker

In comparing patterns of population structure and history inferred from microsatellite and mitochondrial DNA some general patterns have emerged. Overall, there was agreement in the major lineages/clades/biotypes identified from our microsatellite analysis with those obtained from mitochondrial DNA sequences in several studies [19, 20] and from our data herein (S1 Table, Fig 3). With some exceptions, discussed below, the relationships between populations in different continents (e.g., Americas, Asia), as well as host-associated structure (e.g. African cassava–associated populations; the Jatropha-PR biotype (probably once distributed throughout in Caribbean Basin, pre-B biotype invasion) corroborates the herein envisaged worldwide structure of *B*. *tabaci* populations.

Some discrepancies were observed however that suggest the cautious use of mitochondrial DNA as a single marker for delineating species boundaries, and further in describing new species in this group, despite the methodological efficiencies involved [20, 26, 49], because a single marker will not usually provide an accurate picture of population histories. For example, in our microsatellite analysis we found that the population from France we examined clearly clusters with the Western Mediterranean Q biotype (Spain Q), with evidence of gene flow with the Spanish Q-Western Mediterranean population. When the mtDNA of these populations was examined, however, the French population had the Eastern Mediterranean mitochondrial haplotype (the same as Cyprus, Turkey, Israel) [54]. This implies female-biased admixture, between Eastern Mediterranean females and Western Mediterranean males with offspring retaining their maternal mtDNA, but with gene flow evident in their nuclear DNA. While it is possible that the mtDNA of Eastern Mediterranean has a competitive advantage over the Western Mediterranean, the first hypothesis sounds more plausible, as it could be explained with the involvement of *Wolbachia* or *Cardinium* infections. Therefore, a mtDNA phylogeny would have erroneously assigned the French population to the Eastern Mediterranean Q subclade, ignoring the background nuclear gene flow and provide a misleading picture of the species and more recent population histories.

Similar patterns, with conflicting assignments of populations to the Eastern and Western Mediterranean Q subclades between the microsatellite and mitochondrial DNA were observed in other populations we examined (Q biotype-Greece and S biotype–Spain) indicating that this was not an isolated case [54]. In fact, similar findings were also interestingly reported by another study [50], which showed that the Western Q1 and Eastern Q2 mitochondrial haplotypes were associated with different nuclear backgrounds, with evidence for admixed nuclear patterns in samples from France. The high prevalence of *Wolbachia*, *Cardinium*, *Rickettsia* and other endosymbiont infections in *B*. *tabaci* [38, 50, 52, 126, 131–133] suggests that impacts of symbionts are common phenomenon in this insect. Furthermore, the influence of endosymbionts might be even more profound if populations are infected with different bacterial strains or species, as was found by a number of studies in populations of the Western Q1 and Eastern Q2 across the Mediterranean [50, 51, 115]. Several lines of evidence suggest that maternally inherited endosymbionts can have huge effects on mtDNA evolution, by either decreasing inter-population genetic diversity (with a symbiont-induced, selective sweep and mtDNA hitchhiking along) or by increasing diversity following fixation of different symbiont strains in different populations (which would show high haplotype differentiation, despite ongoing nuclear gene flow which would remain unchanged) [34].

The possible effects of endosymbiont composition in the evolution of the mtDNA lineages in *B*. *tabaci* are discussed in Gueguen et al [38], who suggest that disentangling the associations between mtDNA evolution and endosymbiont community would require a comparison between the molecular evolution of mitochondrial markers and that of suitable nuclear markers. Moreover, the authors stress out that the close association between mitochondrial diversity and endosymbiont community may render mtDNA markers unsuitable for biotype identification and phylogeny and should be taken into account when attempting to define biotype or species boundaries [38]. Furthermore, other properties of the mtDNA, such as the reduced effective population size of the marker and introgression, maternal inheritance, recombination, inconsistent and complex rates of mutation, its location in a metabolically active, highly oxidative environment, and heteroplasmy [33, 35] may have very strong effects in the organisms’ evolutionary history, which may confound the interpretation and reconstruction of its phylogeny.

Another case of conflict between nuclear-mitochondrial data is in the genetic relationships among biotypes. For example, in this study the Ms biotype is more closely related to the B biotype-like subclade, including its relative from Yemen, based on the observation that the two biotypes shared more multilocus genotypes compared to either of the two with the Q biotype. This result is in agreement with that obtained for the nuclear ITS1 gene phylogeny [38] in which the two biotypes shared 100% nt sequence identity for this nuclear marker. However, in the latter study the mtCOI phylogeny showed that the Ms biotype was more closely related to Q than to the B biotype, which conflicts other published mtCOI phylogenies showing that B and Q biotypes are sister clades, while Ms is an outgroup [20, 42]. These discrepancies can only be resolved with further analysis of multiple markers from different parts of the genome combined with other data, but they certainly indicate the degree of uncertainty and error we encounter by relying to a single gene phylogeny for delineating relationships within this group.

The results of this study demonstrate that despite their cryptic nature, *B*. *tabaci* entities (biotypes or haplotypes) represent a collection of very old lineages, perhaps isolated from each other for millions of years as has been suggested for other cryptic taxa. The analysis using nuclear microsatellite markers from populations worldwide confirms extreme levels of genetic differentiation in the nuclear genome. Although the pattern of genetic/geographic structure is generally consistent to that obtained from the mtCOI phylogeny, some examples were identified in which there is a strong conflict between the two markers. In this light caution should be taken when using mtDNA as a single marker for phylogenetic reconstruction and species characterization for *B*. *tabaci*. Analysis of multiple genetic markers from different parts of the genome are likely better able to aid in corroborating biological (phenotypic) differences in populations, provide information about the relative time of divergence among biotypes, and identify loci under selection. Thus, a multifaceted approach that incorporates biological, ecological, and behavioral studies, crossing experiments, and better knowledge of the role of endosymbiont contributions to the obstruction of gene flow, together with genetic information is needed to understand and determine species boundaries in this complex sibling species group.

## Supporting Information

S1 Fig Plot showing the number of genetic clusters *K* against the Δ*K* estimator derived from STRUCTURE HARVESTER using the Evanno et al method. (TIF)

S2 Fig Plot showing the number of likely genetic clusters (*K*) against the estimated Ln probability of data. (TIF)

S3 Fig Bayesian clustering analysis results of worldwide multilocus genotypes of *B*. *tabaci* performed in Structure showing clustering results at *K* = 8. (TIF)

S4 Fig Bayesian clustering analysis results of worldwide multilocus genotypes of *B*. *tabaci* performed in Structure showing clustering results at *K* = 10. (TIF)

S5 Fig Bayesian clustering analysis results of worldwide multilocus genotypes of *B*. *tabaci* performed in Structure showing clustering results at *K* = 11. (TIF)

S6 Fig Bayesian clustering analysis results of multilocus genotypes of *B*. *tabaci* biotype Q populations (Eastern and Western Mediterranean) performed in Structure. (TIF)

S1 Table Whitefly collections used in this study with collection and population/affiliated biotype information. ^a^Samples with the same number were collected from neighboring locations and were pooled together for analysis after exact tests of population differentiation showed non-significant differentiation. ^b^Population names refer to the geographic sampling location and were given a biotype alphabetic code (e.g. A, B) if they originated from a reference collection previously characterized. ^c^GenBank accession number for mtCOI sequence of one individual from each of the populations sampled in this study. ^d^Number of adult females genotyped. * Populations that were excluded from the PCA analysis.
(XLSX)

S2 Table Locus specific statistics across populations. ^a^Number of alleles. ^b,c^Mean expected (*H*_E_) and observed (*H*_O_) heterozygosities (averaged across populations) (±SE). ^d^Inbreeding coefficient within individuals relative to the population (*F*_IS_). ^e^Number of populations that deviated from HWE in each locus out of a total of 41 populations
(XLSX)

S3 Table Population specific statistics for each of the 13 loci genotyped. ^a^Samples collected from neighboring locations, which showed non-significant differentiation (exact tests of population differentiation) were pooled together, as shown in S1 Table. ^b^Number of different alleles over loci. ^c,d^Expected (*H*_E_) and observed (*H*_O_) heterozygosities over loci. ^e^Mean fixation index (*F*) over loci (±SE). ^f^Estimate of null allele frequency using the EM algorithm as described in Materials and Methods.
(XLSX)
