# Supplementary figures and images for: Global Population Structure of a Worldwide Pest and Virus Vector: Genetic Diversity and Population History of the Bemisia tabaci Sibling Species Group
Source: PLoS One. 2016 Nov 17;11(11):e0165105. doi: 10.1371/journal.pone.0165105 (PMC5113902; doi:10.1371/journal.pone.0165105)

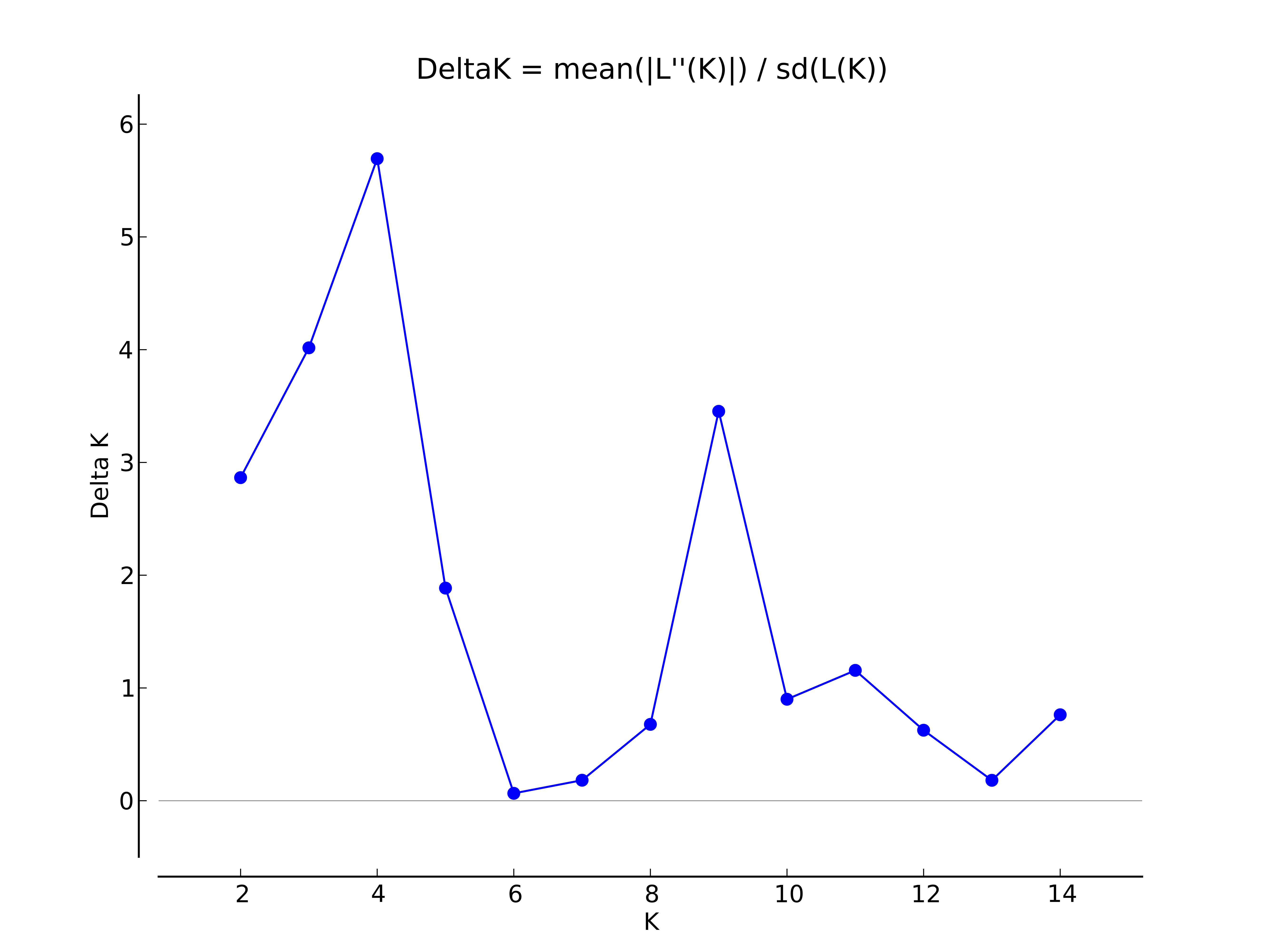

Supplement: S1 Fig — (TIF) [file pone.0165105.s001.tif]

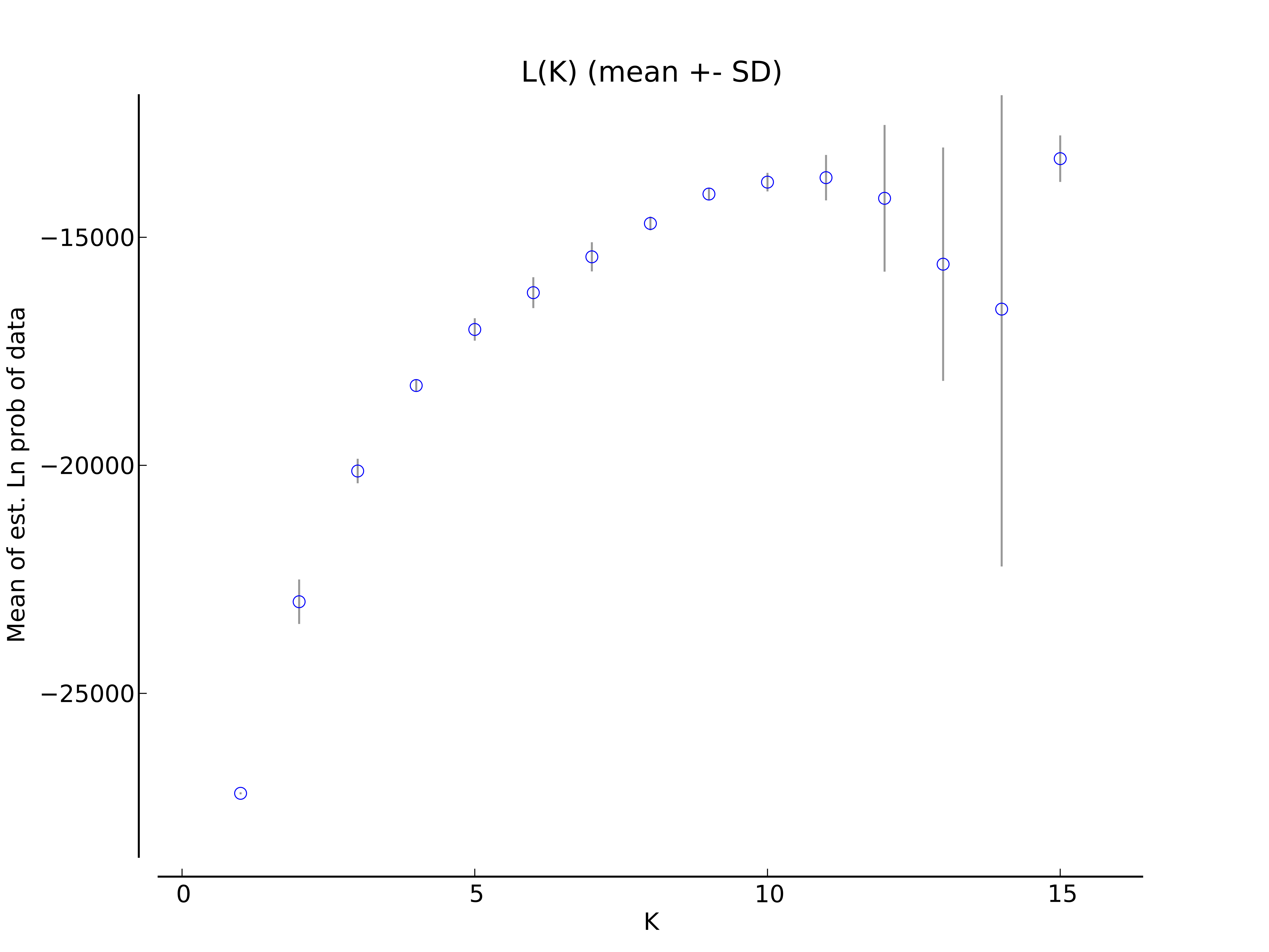

Supplement: S2 Fig — (TIF) [file pone.0165105.s002.tif]

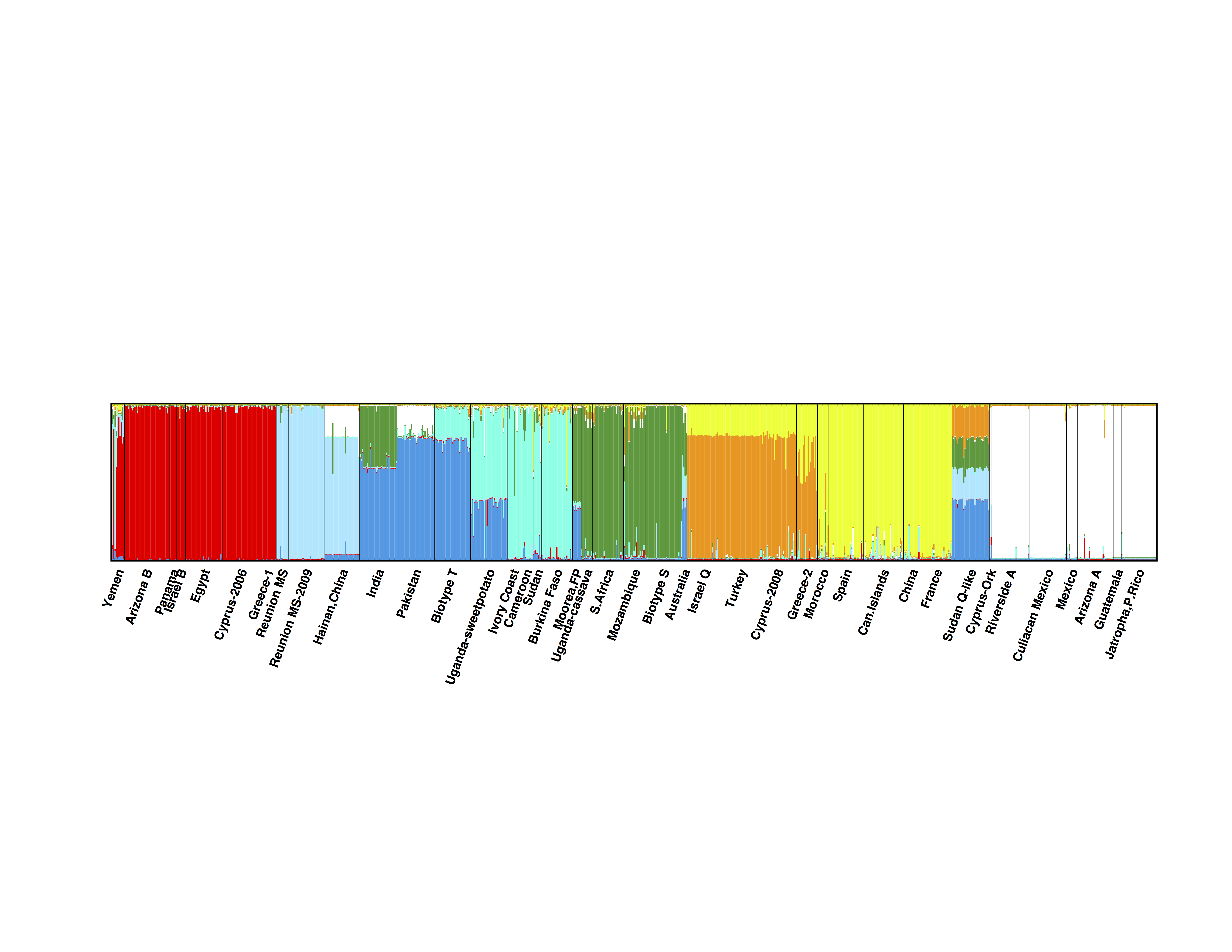

Supplement: S3 Fig — (TIF) [file pone.0165105.s003.tif]

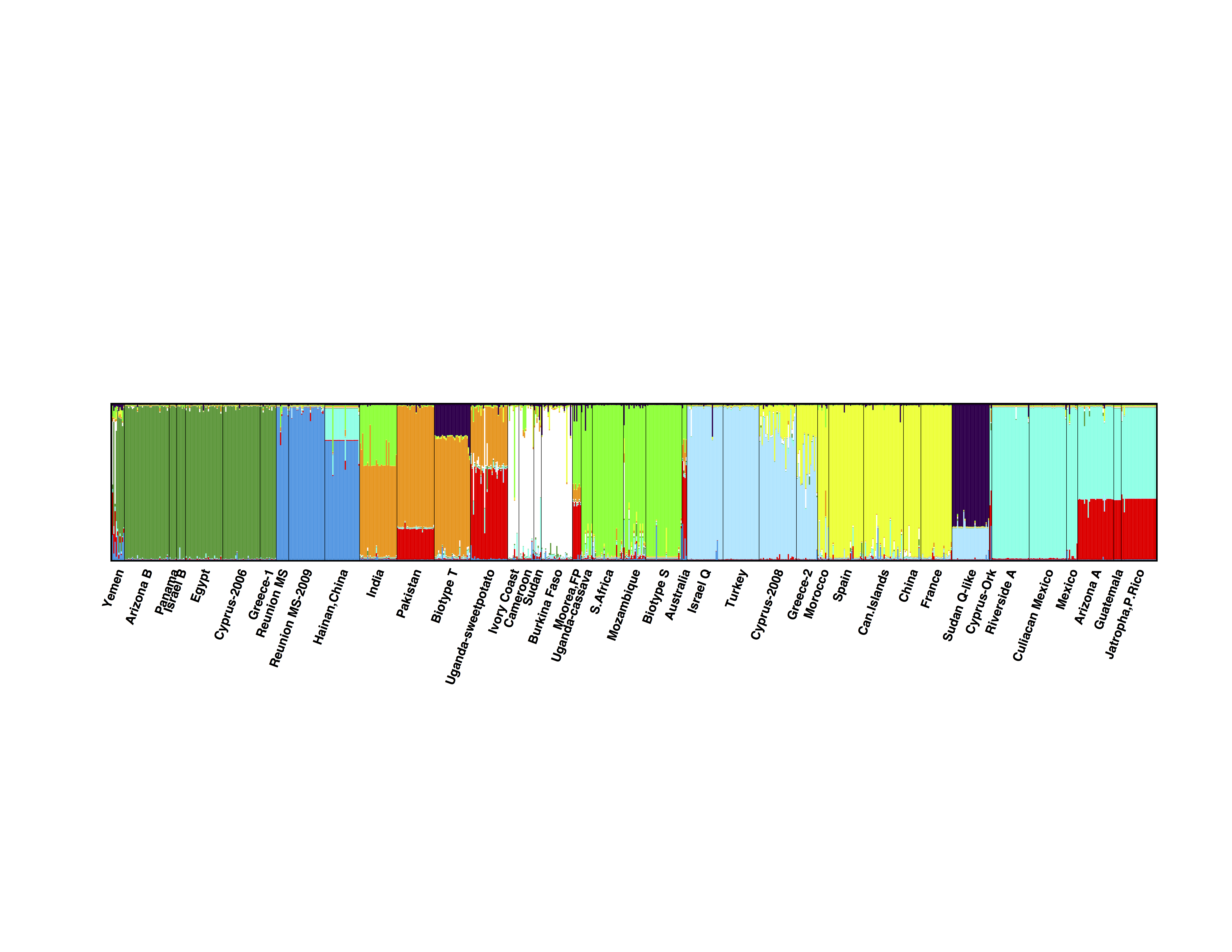

Supplement: S4 Fig — (TIF) [file pone.0165105.s004.tif]

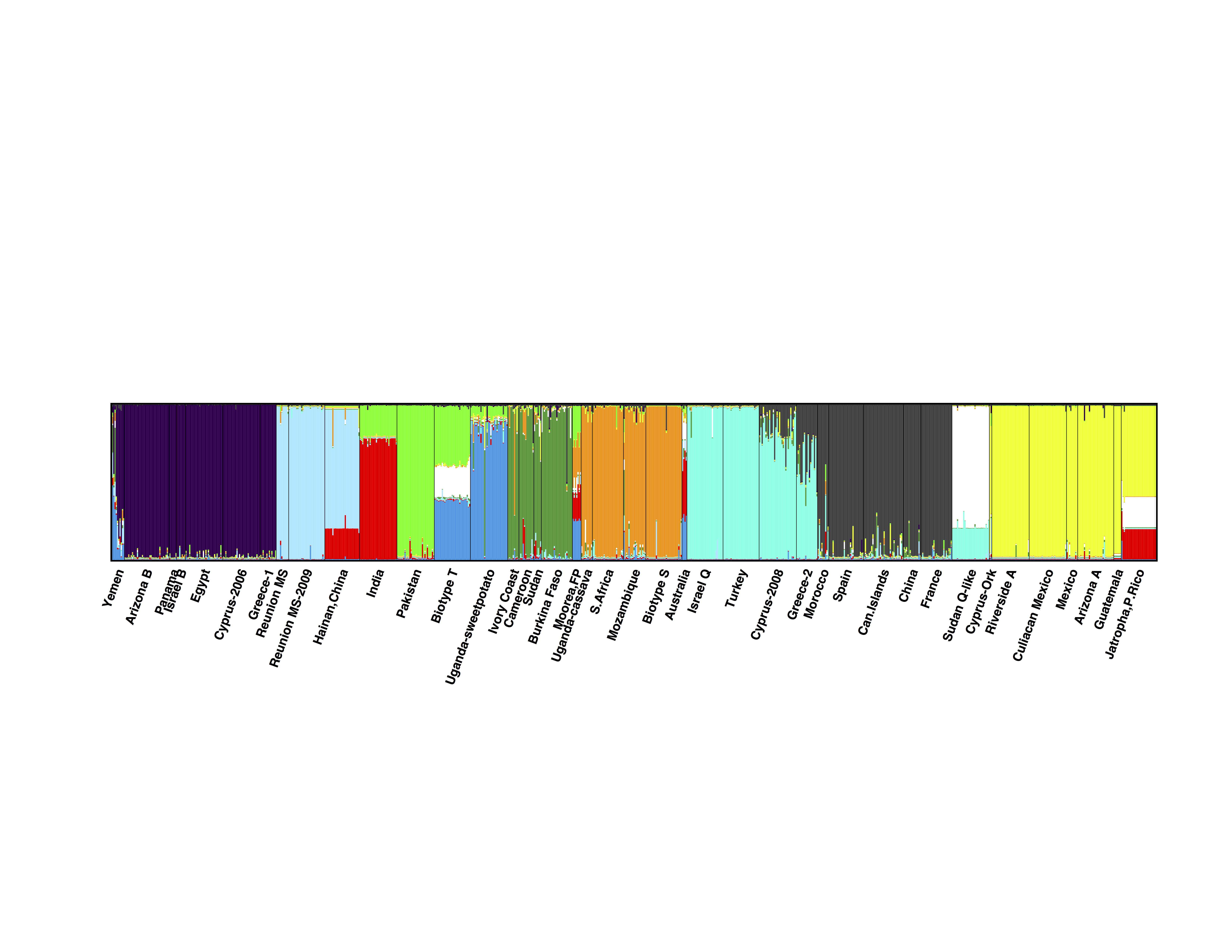

Supplement: S5 Fig — (TIF) [file pone.0165105.s005.tif]

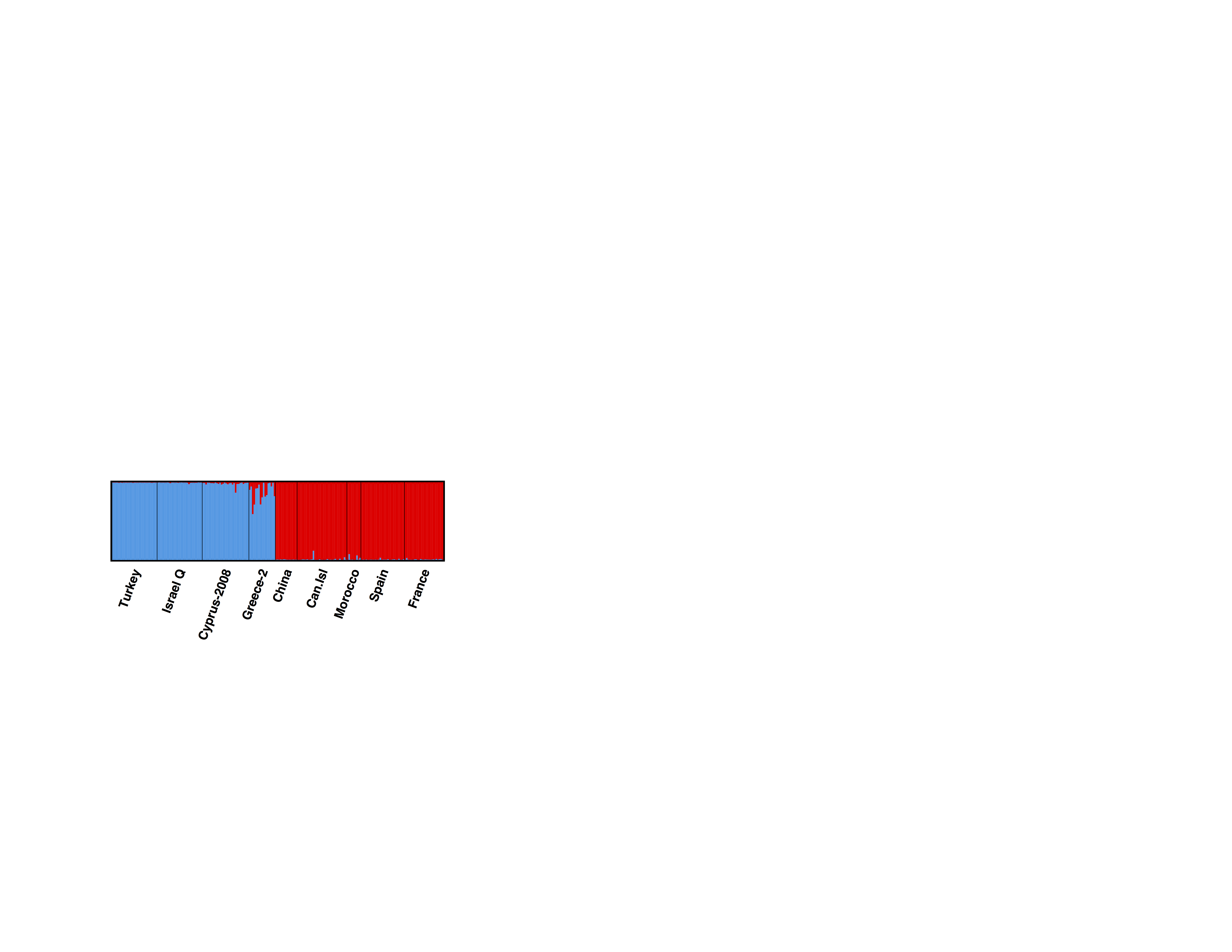

Supplement: S6 Fig — (TIF) [file pone.0165105.s006.tif]
